# T-lymphoid progenitor-based immunotherapies: clinical perspectives for one and all

**DOI:** 10.1038/s41423-022-00927-5

**Published:** 2022-09-30

**Authors:** P. Gaudeaux, R. D. Moirangthem, J. Paillet, M. Martin-Corredera, H. Sadek, P. Rault, A. Joshi, J. Zuber, T. S. Soheili, O. Negre, I. André

**Affiliations:** 1 Laboratory of Human Lymphohematopoieisis, Imagine Institute, INSERM UMR 1163, Université Paris Cité, Paris, France; 2 Smart Immune, Paris, France

**Keywords:** Allotransplantation, Lymphopoiesis

Commentary of Moirangthem, R.D., Ma, K., Lizot, S. et al. A DL-4- and TNFα-based culture system to generate high numbers of non-modified or genetically modified immunotherapeutic human T-lymphoid progenitors. Cell Mol Immunol 18, 1662–1676 (2021).

Here, we present a discussion on the use of human T lymphoid progenitors (HTLPs) as a platform for cell and gene therapy, enabling promising strategies to deal with unmet medical needs beyond oncology, such as autoimmunity and organ transplantation. We also comment on the intrinsic potential of HTLPs to be expanded, educated, and tolerized by the recipient’s thymus as a first-in-class “off-the-shelf” T-cell progenitor therapy accessible to a large number of patients.

T lymphocytes are key actors of the adaptive immune system, which plays an instrumental role in the defense against infectious agents and malignant cells. The medical breakthrough achieved with the rapid development of chimeric antigen receptors (CARs), especially in the field of oncology, has sparked renewed interest in T-cell function and homeostasis. “Off-the-shelf” strategies offer a promising approach to addressing the high manufacturing costs and limited accessibility of advanced therapy medicinal products such as CAR-T cells. However, cell compatibility as well as time-limited efficacy and accelerated exhaustion are challenges hampering the democratization of “off-the-shelf” T-cell-based therapy biobanking.

CAR-engineered T cells are redirected toward target cells through the recognition of a cognate antigen without any MHC restriction. In addition to its use in oncology, CAR engineering is opening up new therapeutic avenues in the fields of organ transplantation, autoimmunity and infectious diseases [1, 2].

However, the widespread use of CAR-T cells still faces some challenges, including time-limited efficacy due to their accelerated exhaustion after administration. Early functional loss following CAR-T-cell transfer primarily results from the in vitro manufacturing procedure, based on supra-physiological stimulation. Furthermore, the potential to promote terminal differentiation can be further enhanced by CAR tonic signaling [3]. To be effective, CAR-T cells must be injected in high doses (dose escalation clinical trials have used a typical range of 10^6^–10^9^ CAR T cells per patient) with significant consequences in terms of manufacturing cost and exposure of patients to this disruptive therapeutic strategy.

Allogeneic CAR-T cells may allow the treatment of several patients from one manufacturing batch and provide readily available cellular products for patients. Unfortunately, this promising approach suffers from several drawbacks: (1) risks of graft-versus-host disease (GvHD) and rejection; (2) the necessity to perform multiple edits to the genome (T-cell receptor (TCR) and human leukocyte antigen (HLA)), which increases the complexity of the procedure and the risk of chromosome instability; (3) the necessity to inject very high doses of cells; and (4) the short persistence of the cells in vivo.

The use of CAR-engineered human T lymphoid progenitors (CAR-HTLPs) from allogeneic donors or from induced pluripotent stem cells (iPSCs) could address these issues. Tolerized allogeneic naïve T cells expressing a CAR could be generated after differentiation and education of these CAR-HTLPs in the thymus of the patients. CAR-HTLPs may benefit from the host’s capacity to sustain their proliferation during their thymus differentiation, maturation, and further activation, which should allow treatment efficacy with a low dose of cells.

A process enabling the ex vivo production of HTLPs from CD34^+^ hematopoietic stem and progenitor cells (HSPCs) was initially developed to address prolonged T-cell lymphopenia following allogeneic hematopoietic stem cell transplantation (alloHSCT). HTLPs are able to colonize the thymus directly without homing to the bone marrow, initiating rapid thymopoiesis following their transplantation. We recently demonstrated the critical role played by TNFα in synergy with the Notch pathway during HSPC differentiation, with TNFα increasing cell proliferation and leading to an efficient scale-up of cell manufacturing [4]. We also developed a protocol enabling efficient gene modification of HTLPs with a CAR construct, providing new opportunities for the establishment of lymphoid progenitor-based therapies such as CAR-HTLPs [4, 5].

Here, we explore promising strategies based on the use of CAR technology combined with HTLPs to address unmet medical needs. We also discuss the ability to generate “off-the-shelf” lymphoid progenitor-based therapies through straightforward protocols using new sources of cells and with improved manufacturing yields.

The development of CARs has provided promising application opportunities beyond oncology in infectious diseases, organ transplantation, and autoimmunity [1]. The possibility of obtaining CAR-expressing HTLPs with high yields and purity from a small number of genetically modified HSPCs [4, 5], which should in turn give rise to a high number of mature CAR T cells after their maturation in the recipient’s thymus, has created new opportunities for the development of therapeutic strategies.

Such an approach would significantly decrease the quantity of vectors and other GMP reagents required to obtain an equivalent number of genetically modified mature T cells. Another advantage relies on the lack of expression of any T-cell receptor by HTLPs, which, combined with their education in the host thymus, prevents any graft-versus-host reaction and enables the generation of “off-the-shelf” allogeneic products without editing the TRAC. Using cells from healthy donors avoids manufacturing products from cells that were exposed to chemotherapies with alkylating agents. Finally, the production of naïve T cells after HTLP infusion addresses the time-limited efficiency observed with mature overactivated CAR-T cells.

However, the use of HTLPs to produce CAR-T cells faces several challenges that need to be addressed. Very recently, van der Stegen and colleagues discussed the dramatic impact of early CAR expression in lymphoid progenitors during their maturation into T cells: a constitutively expressed CAR in T-cell-derived iPSCs was shown to promote the acquisition of an innate phenotype, generating cells that were transcriptionally closer to γδTCR-T cells than αβTCR-T cells. The researchers further demonstrated that premature αβTCR or constitutive CAR expression interferes with double-positive cell maturation, depending on the strength of Notch stimulation. They bypassed this difficulty on the one hand by delaying CAR expression through TRAC promoter-controlled expression and on the other hand by calibrating CAR signaling through CD3ζ ITAM mutations [6].

Wang and colleagues obtained mature and functional CAR-T cells from iPSCs [7] using a 3D artificial thymic organoid (ATO) system. They indicated the importance of selecting T_n_/T_mem_-derived iPSCs, which resulted in a more homogeneous, monoclonal TCR repertoire different from the polyclonal phenotype of iPSCs reprogrammed from CD34^+^ cord blood HSPCs. The researchers postulated that starting with a less differentiated T_n_/T_mem_ population may have a specific effect on TCR rearrangement during redifferentiation.

These two recent studies highlight the impact of the expression of “active molecules”, such as CARs, on cell differentiation toward the T lineage: the timing of expression appears especially critical to not impair cell integrity during maturation. In this regard, implementing gene modification of HTLPs ahead of thymic selection steps could have unwanted downstream effects on cell functionality and should be investigated carefully. Regulatable CAR gene expression systems are currently being explored and could solve such a problem.

The potential of HTLPs to give rise to a complete T-cell compartment, including all T-cell subsets, makes it possible to consider their use to produce CAR-Tregs. FOXP3-expressing CD4^+^ CD25^+^ T cells (hereafter referred to as Tregs) are broadly recognized as the physiological guardians of tolerance and are endowed with the capabilities to suppress immune responses against self-antigens, alloantigens (during pregnancy), food antigens, and bacteria from the commensal flora [8]. There is thus tremendous interest in harnessing Tregs for the induction or restoration of immune tolerance. However, the low frequency of Tregs with antigen-specific reactivity may account for the reportedly limited impact of polyclonal Treg therapies in the fields of autoimmunity and transplantation.

In this respect, CAR-engineered Tregs, redirected toward an organ-specific antigen, hold much promise to protect the organ from an immune assault [2]. However, we previously showed that the manufacturing of CAR-Tregs from highly purified naïve Tregs required dramatic in vitro expansion over a cumbersome and costly 2- to 3-week culture, which could destabilize Tregs, shorten their in vivo lifespan, and mitigate their suppressive function [3]. The generation of CAR-Tregs from a large population of human naïve T cells, converted to induced Tregs with a cocktail of cytokines, could theoretically address this issue by reducing the need for in vitro expansion. However, all attempts to induce in vitro stable human Tregs locked in their identity through a reinforcing loop have failed. No ex vivo protocol has been able to recapitulate the complexity of bona fide Treg thymic ontogeny, which is initiated by early chromatin remodeling and, thought to result from instructive (high avidity TCRs) and stochastic factors (IL-2, CD80, and TNFR ligand availability) [8].

The use of HTLPs engineered to express a CAR in a Treg-specific manner would overcome all the abovementioned obstacles and leverage the fantastic physiological expansion of thymocytes without jeopardizing Treg stability. A recent study from Stanford’s group might be a game changer in this respect [9]. The expression of a transgene was successfully driven by *FOXP3* regulatory elements so that only the Tregs generated in vivo from genetically modified hematopoietic stem cells expressed the transgene. This thrilling pioneer study laid the groundwork for a new era of regulatory cell therapies derived from T-cell progenitor engineering.

Expanding the clinical applications of the HTLP platform as discussed is not feasible without an “off-the-shelf” strategy. In addition to primary cells having been investigated for the production of HTLPs [4, 5], iPSCs, which can be easily genetically modified and are endowed with self-renewal capacities, may also constitute another source for “off-the-shelf” HTLPs. Notably, clonal selection can guarantee homogeneous cell product batches [10]. Attempts to supply T-cell immunotherapies with “off-the-shelf” T cells have fueled active and productive research. Different groups have provided evidence of the successful generation of highly proliferative T cells from iPSCs through the reprogramming of mature T cells while retaining expression of the rearranged TCR [11]. A few studies have used hDL-1- or hDL-4-expressing MS5 or OP9 feeder cells to produce CD7^+^ T-cell progenitors from iPSCs [7]. However, the use of murine-derived feeder cell-based culture systems creates obstacles for their translation into clinical applications.

**Fig. 1:**
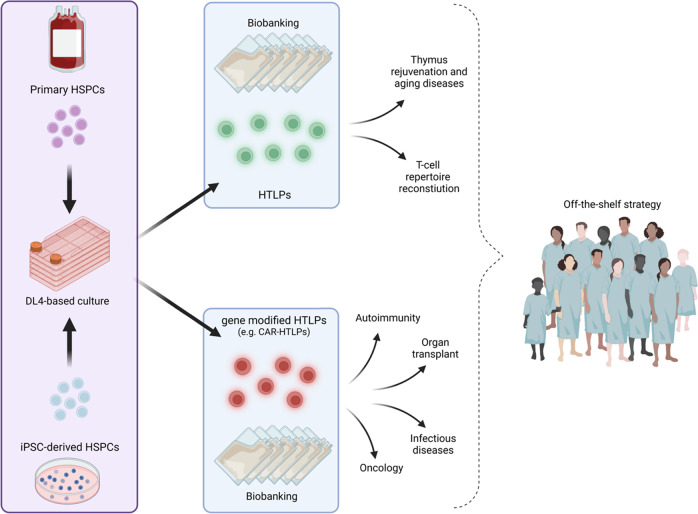
Manufacturing of human T lymphoid progenitors, both from primary cells and from iPSCs, enables the development of new therapeutic strategies with or without gene modification of starting cells with the aim of generating biobanks compatible with “off-the-shelf” therapy targeting the largest number of patients. Created with BioRender.com

Therefore, significant efforts have been made over the past years to develop clinical-grade reagents and procedures for the generation of T-cell progenitors and T cells from iPSCs [11]. Nevertheless, the safety issues associated with iPSC-based cell therapy cannot be overlooked. Genetic and epigenetic abnormalities occurring during the reprogramming and/or subsequent culture of iPSCs have been reported. Such genetic drift may initiate malignant transformation and generate immunogenicity and variability across iPSC lines and batches [12]. Therefore, on the path toward clinical translation, standardized quality control of iPSCs and their derivatives is mandatory and warrants further studies (Fig. 1).

In line with a potential “off-the-shelf” strategy, HLA compatibility and HLA-associated barriers should be considered. Only 30% of patients eligible for alloHSCT can benefit from a transplantation from an HLA-matched sibling donor as the best source in this clinical setting. As mentioned above, HTLPs do not express a TCR and consequently are unable to mediate GvHD. Zakrzewski et al. showed that adoptive transfer of allogeneic T-cell progenitors to irradiated mice in a fully mismatched MHC context was a safe and potent strategy to give rise to recipient-restricted donor-derived T cells devoid of graft-versus-host reactivity [13]. Furthermore, the study demonstrated the capacity to maintain the graft-versus-leukemia effect, despite the “pruning” of alloreactive T cells during thymic ontogeny, through the transgenic expression of a tumor-specific CAR. These results stress the possibility of developing therapeutic strategies based on “off-the-shelf” manufacturing of universal T-cell progenitors with more flexibility in the consideration of HLA mismatch.

We demonstrated that in our TNFα-supplemented immobilized DL-4 culture system, CB-derived HTLPs had increased expression of insulin-like growth factor 2 mRNA-binding proteins (IGF2BP1 and IGF2BP3), which are implicated in cell survival and proliferation, compared to mPB-derived HTLPs [5]. This is in accordance with the fact that the yield and purity of HTLPs were higher with CB-isolated HSPCs than with those isolated from mPB. The quantity of manufactured HTLPs derived from one CB unit may be sufficient to treat at least five patients. Furthermore, in the HSCT setting, CB-isolated HSPCs are known to require less stringent HLA compatibility than mPB-isolated HSPCs [14].

Multiple batches of CB-isolated CD34^+^ HPSC-derived immunotherapeutic HTLPs could be produced from CB samples and subsequently cryopreserved and banked as “ready to use” cell products. The banking of “universal” CB- or iPSC-derived HTLPs or of a few haplotypes would address the issue of cell manufacturing in a timely manner for severely ill patients and would dramatically shorten the time between treatment decision and treatment delivery.
