# Author Correction to: T-lymphoid progenitor-based immunotherapies: clinical perspectives for one and all

**DOI:** 10.1038/s41423-022-00935-5

**Published:** 2022-11-14

**Authors:** P. Gaudeaux, R. D. Moirangthem, J. Paillet, M. Martin-Corredera, H. Sadek, P. Rault, A. Joshi, J. Zuber, T. S. Soheili, O. Negre, I. André

**Affiliations:** 1 Laboratory of Human Lymphohematopoieisis, Imagine Institute, INSERM UMR 1163, Université Paris Cité, Paris, France; 2 Smart Immune, Paris, France

**Keywords:** Allotransplantation, Lymphopoiesis

Correction to: *Cellular & Molecular Immunology* 10.1038/s41423-022-00927-5, published online 30 September 2022

In this article the affiliation 2 Smart Immune, Paris, France for P. Gaudeaux was missing.

The original article has been corrected.

